# Lower versus Higher Oxygen Concentration for Delivery Room Stabilisation of Preterm Neonates: Systematic Review

**DOI:** 10.1371/journal.pone.0052033

**Published:** 2012-12-20

**Authors:** Jennifer V. E. Brown, Thirimon Moe-Byrne, Melissa Harden, William McGuire

**Affiliations:** 1 Centre for Reviews and Dissemination, University of York, York, United Kingdom; 2 Hull York Medical School, University of York, York, United Kingdom; The Ohio State Unversity, United States of America

## Abstract

**Background:**

Emerging evidence suggests that initiating delivery room respiratory support or resuscitation for term infants using lower rather than higher concentrations of oxygen reduces mortality and the risk of serious morbidity. Uncertainty exists with regard to applicability of this strategy for preterm infants who have different underlying reasons for respiratory distress and risks for harm at birth than term infants.

**Methods:**

We performed a systematic review and meta-analysis of randomised controlled trials to determine the effect on mortality and morbidity of using lower (21– 50%) versus higher (>50%) oxygen concentrations for delivery room transition support of preterm infants.

**Results:**

We identified six randomised controlled trials in which a total of 484 infants participated. Most participants were preterm infants born before 32 weeks’ gestation. One trial was quasi-randomised and in one trial allocation concealment was not described. Clinicians and investigators were aware of the interventions in all but one trial. Meta-analyses found a statistically significant reduction in the risk of death pooled risk ratio 0.65 (95% confidence interval 0.43, 0.98), but this effect disappeared when only the four trials with adequate allocation concealment were included [pooled risk ratio 1.0 (95% confidence interval 0.45, 2.24)]. None of the trials has evaluated any neuro-developmental outcomes.

**Conclusions:**

The available trial data do not provide strong evidence that using lower versus higher oxygen concentrations for delivery room transition support for preterm infants confers important benefits or harms. Lack of allocation concealment and blinding of clinicians and assessors are the major sources of bias in the existing trials. Further, large, good-quality trials are needed to resolve on-going uncertainties and inform clinical practice.

## Introduction

Respiratory complications of preterm birth are an important cause of infant mortality and morbidity. Primarily, respiratory distress syndrome in preterm infants is due to deficiency of pulmonary surfactant, a complex mixture of phospholipids and proteins that reduces alveolar surface tension and maintains alveolar stability. As most alveolar surfactant is produced after about 32 weeks’ gestation, very preterm infants born before then are at high risk of developing respiratory distress syndrome.

Very preterm infants who have delayed establishment of independent respiratory effort after birth may require delivery room transition support including positive pressure ventilation and oxygen supplementation. Concern exists that excessive positive pressure ventilation and exposure to high oxygen concentrations may be harmful to very preterm infants. Recent updates of international consensus guidelines have advocated a less invasive approach to respiratory support and stabilization [1], [2]. These include using initial lower concentrations of oxygen (including air) rather than 100% oxygen during initial respiratory support and titrating any increase in oxygen concentration to clinical response. Because clinical assessments of oxygenation are inaccurate in very preterm infants receiving transitional support, guidelines also advocate the use of pulse oximetry to guide respiratory interventions.

The recommendations to restrict use of high oxygen concentrations have been informed mainly by evidence from trials in which most participants were term or near-term infants who required transition support after birth because of perinatal asphyxia. In this clinical context, evidence from controlled trials suggests that respiratory support using lower concentrations of oxygen (including air) may reduce mortality and morbidity [3], [4], [5], [6]. It is postulated that even brief exposure to high oxygen concentrations after birth may trigger pathogenic cascades for reperfusion and re-oxidation damage [7].

It is unclear to what extent this evidence is applicable to preterm infants. Although potentially more susceptible to reactive oxygen species-mediated reperfusion cytotoxicity, in general very preterm infants are not born following a severe perinatal asphyxial insult. Most very preterm infants establish respiratory effort and attain oxygen saturation levels without active respiratory resuscitation [8], [9]. However, many infants, especially extremely preterm infants, require supplemental oxygen during transition support to attain recommended target oxygen saturations and hyperoxia may have specific therapeutic advantages in newborn infants with pulmonary hypertension [10]. It is noteworthy that a recent meta-analysis of good-quality trials found that mortality was higher in extremely preterm infants in who lower oxygen saturation levels were targeted during care in the neonatal unit following admission from the delivery room [11].

Given this potential for the level of oxygen administration to have both harmful and beneficial effects for preterm infants receiving delivery room transition support, we have undertaken a systematic appraisal and review of randomised controlled trials that assessed this intervention in order to determine implications for current practice and future research.

## Methods

We conducted a systematic review of randomised controlled trials using the standard methods of the Cochrane Neonatal Review Group and the NIHR Centre for Reviews and Dissemination [12], [13]. We registered the protocol on PROSPERO, the international prospective register of systematic reviews (registration number CRD42012001906). We adhered to the conduct and reporting guidelines suggested in the “Preferred Reporting Items for Systematic Reviews and Meta-Analyses (PRISMA)” statement [14] (Table S1).

### Search Strategy

We searched the following electronic databases: MEDLINE, MEDLINE In-Process & Other Non-Indexed Citations, EMBASE, Maternity and Infant Care, CINAHL, Cochrane Database of Systematic Reviews (CDSR), Database of Abstracts of Reviews of Effects (DARE), Health Technology Assessment Database (HTA), Cochrane Central Register of Controlled Trials (CENTRAL), ClinicalTrials.gov, Current Controlled Trials, WHO International Clinical Trials Registry Platform (ICTRP). We did not apply any language restrictions or date limits. We used a validated search filter, where available, to limit retrieval to clinical trials. The full search strategies and results for each database are described in appendix S1.

We searched the abstracts from the annual meetings of the Pediatric Academic Societies (1993 to 2012), the European Society for Pediatric Research (1995 to 2012), the UK Royal College of Paediatrics and Child Health (2000 to 2012), and the Perinatal Society of Australia and New Zealand (2000 to 2012). We considered trials reported only as abstracts to be eligible if sufficient information was available from the report, or from contact with the authors, to fulfill the inclusion criteria.

We searched the following web sites for guidelines on neonatal resuscitation: National Guideline Clearinghouse, National Institute of Health and Clinical Excellence (NICE), Scottish Intercollegiate Guidelines Network (SIGN), Turning research into practice database (TRIP), Resuscitation Council (UK), European Resuscitation Council, Royal College of Paediatrics and Child Health, Royal College of Obstetricians and Gynaecologists, Royal College of Midwives and the British Association of Perinatal Medicine.

We searched the bibliographies of all relevant reviews, guidelines and included studies.

### Study Selection

Inclusion criteria are summarised in table 1. Two reviewers independently screened titles and abstracts of all records identified in the search and ordered full papers for any potentially relevant trials. The full texts were re-assessed and those studies that did not meet all of the inclusion criteria were excluded. Any disagreements were discussed with a third reviewer until consensus was achieved.

**Table 1:** Inclusion Criteria.

**Design**	Randomised or quasi-randomised controlled trials
**Participants**	Preterm (<37 weeks) or low birth weight (<2.5 kg) infants
**Intervention**	Low oxygen concentration (21–50%)
**Comparison**	High oxygen concentration (>50%)
**Co-intervention**	Monitoring of oxygen levels by pulse oximetry (optional)

### Data Extraction and Quality Assessment

Two reviewers used piloted data extraction forms to collect basic study information and details on participants, treatment, and control interventions, as well as outcome data as specified in table 2. We used the Cochrane Risk of Bias tool to independently assess the methodological quality of any included trials in terms of selection bias, performance bias, detection bias, and attrition bias [15]. Additional information from the trial authors was requested to clarify methodology and results as necessary. Any disagreements in data extraction were resolved by consensus in discussions between three reviewers.

**Table 2:** Outcomes.

*Primary*	All-cause mortality prior to hospital discharge
	Neurodevelopmental outcomes assessed using validated tools at >12 months post-term, classifications of disability, and cognitive and educational outcomes at >5 years
*Secondary*	Apgar score up to 10 minutes after birth
	Receipt of endotracheal intubation
	Receipt of surfactant replacement
	Proportion of infants reaching the target oxygen saturation range (defined by authors) within 10 minutes
	Chronic lung disease (CLD) or bronchopulmonary dysplasia (BPD
	Retinopathy of prematurity (ROP)
	Necrotising enterocolitis (NEC)
	Severe intraventricular haemmorhage (IVH grade III/IV)
	Duration of mechanical ventilation
	Duration of supplemental oxygen therapy
	Duration of hospital stay (days)

### Analysis

We performed meta-analyses using the fixed effect model in the Cochrane Collaboration RevMan 5.1 software. We calculated risk ratio (RR) and risk difference (RD) for dichotomous data and weighted mean difference (WMD) for continuous data with respective 95% confidence intervals (CIs). We calculated the number needed to treat (NNT) for a statistically significant reduction in the pooled risk difference.

We examined the treatment effects of individual trials and heterogeneity between trial results by inspecting the forest plots. The impact of heterogeneity in any meta-analysis was assessed using the I^2^ statistic [16]. If statistical heterogeneity was noted, we explored the possible causes using post-hoc sensitivity analyses.

### Analysis of Subgroups or Subsets

We planned these subgroup analyses:

1. Trials in which participants were very preterm (gestational age at birth <32 weeks)
2. Trials undertaken in low or middle-income versus high income countries

## Results

**Figure 1:**
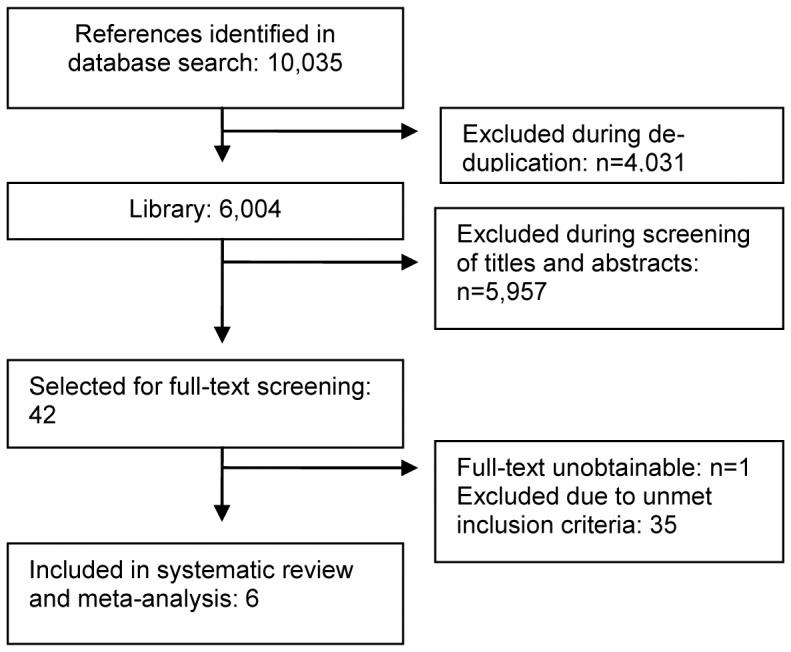
Study flow through the selection process.

Figure 1 illustrates the flow of trials through the selection process. After de-duplication, 6004 records in total were identified through the search. Of those, six trials met all inclusion criteria and were included in the meta-analysis [17]–[22]. These are described in table 3. Two potentially relevant on-going trials were identified [23], [24]. The studies excluded during full text screening are summarised in table S2.

**Table 3:** Characteristics of included studies.

Study(year)	Setting	Method	Participants	Comparisons	Oxygen adjustment criteria	Outcomes
Harling(2005) [17]	Single centre;Liverpool, UK	RCT	<31 weeksgestation	50% (N = 26) versus100% oxygen(N = 26).No routineSpO_2_ monitoring.	**Both groups**: Fixedoxygen concentrationdelivered for duration ofresuscitation until cardiorespiratory stabilityachieved and surfactantgiven.	Death, Apgar score, CLD/BPD, ROP, NEC, need for long-term oxygen therapy
Saugstad(1998) [18]	11 centres inIndia, Egypt,Philippines,Estonia, Spain,Norway	Quasi-RCT(alternatedate ofbirth)	<37 weeksgestation	Air (N = 75) versus100% oxygen(N = 72). Noroutine SpO_2_monitoring.	**Lower group**: Oxygenlevel increased to 100%if infant unresponsiveafter 90 seconds.	Death, Apgar score, proportion of infants reaching the target oxygen saturation All data obtained from authors
Lundstrøm(1995) [19]	Single centre; Copenhagen,Denmark	RCT	<33 weeksgestation	Air (N = 34) versus80% oxygen (N = 6).No routineSpO_2_ monitoring.	**Lower group**: FiO_2_increased in 0.1increments after oneminute in response toheart rate remaining‘below normal’.	Death, Apgar score, receipt of surfactant, ROP, NEC, IVH, need for long-term oxygen therapy
Vento(2009) [20]	2 centres;Valencia, Spain	RCT	≤28 weeksgestation	30% (N = 37) versus90% oxygen(N = 41).	**Both groups**: FiO_2_titrated to achieve targetsaturations, 60–90seconds allowed forresponse after eachchange. If heart rate ≤60beats per minute for>30 s, oxygenconcentration increasedto 100%	Death, Apgar score, receipt of intubation, receipt of surfactant, proportion of infants reaching the target oxygen saturation, CLD/BPD, ROP, NEC, IVH, duration of mechanical ventilation, duration of supplemental oxygen therapy, duration of hospital stay
Wang(2008) [21]	2 centres;San Diegoand Santa Clara,USA	RCT	<32 weeksgestation	Air (N = 18)versus 100%oxygen (N = 23).	**Lower group**: FiO_2_increased to 1.0 ifpersistent bradycardia orchest compression ormedication required.FiO_2_ was increased in0.25 increments ifSpO_2_<70% at 3 min or<85% at 5 min of life**Higher group**:Decreased FiO_2_ at 5 minif SpO_2_>95%	Death, Apgar score, receipt of intubation, receipt of surfactant, IVH, duration of mechanical ventilation
Rabi(2011) [22]	Single-centre;Calgary, Canada	RCT	≤32 weeksgestation	Air (N = 34)versus 100%(N = 72).	**Lower group**: FiO_2_titrated in increments ofup to 0.2 every 15 s toachieve and maintaintarget saturations(85%–92% ).	Death, Apgar score, receipt of intubation, proportion of infants reaching the target O_2_ saturation, CLD/BPD, duration of mechanical ventilation, duration of hospital stay

### Participants

Most included studies were small, single- or two-centre trials conducted since the mid-1990s in Europe and North America. 484 infants participated in total (range 40–147). Most participants were infants born before 32 weeks’ gestation. One study was a multi-centre investigation [18]. This trial recruited both term and preterm infants. Outcome data for the subgroup of preterm infants in this study were provided by the trial investigators for inclusion in this review.

### Interventions

Most of the included trials used room air (21% oxygen) as the “low” oxygen concentration resuscitation gas. Two trials used 30% and 50% oxygen respectively [17], [20]. The most frequently used “high” oxygen concentration gas for resuscitation was 100%. One trial each used 80% and 90% concentrations of oxygen [19], [21].

In the three most recent trials, investigators monitored oxygen saturation levels in all infants and titrated the oxygen concentration of the resuscitation gas accordingly [20]–[22]. In one trial, a subset of infants was monitored using pulse oximetry but it is unclear if this was used as a tool to guide titration of oxygen therapy [18]. The other trials used infants’ heart rate, skin colour, and responsiveness as indicators for changing the oxygen concentration.

One trial contained three randomly allocated groups of participants: a “low” group starting ventilation with 21% oxygen, a “moderate” group starting at 100% oxygen with the option of downward titration depending on the infant’s response, and a “high” group which was ventilated with a static concentration of 100% oxygen (no titration) [22]. For meta-analyses, the “moderate” and “high” groups were combined and treated as a “high” oxygen concentration group.

### Outcomes

All trials reported all-cause in-hospital mortality and Apgar scores during the first 10 minutes after birth. None reported neuro-developmental outcomes. Four trials reported endotracheal intubation or receipt of surfactant replacement. Three trials reported the proportion of infants who reached pre-specified oxygen saturation levels [18], [20], [22]. The incidence of neonatal morbidities including chronic lung disease (CLD) or bronchopulmonary dysplasia (BPD), retinopathy of prematurity (ROP), necrotising enterocolitis (NEC), and severe intraventricular haemorrhage (IVH; grade III/IV) were reported inconsistently (generally by between two and four trials). Two trials reported the length of hospital stay [20], [22].

### Risk of Bias in Included Studies

Most of the trials had some methodological weaknesses (table 4). The two older studies were at unclear and high risk, respectively, of selection bias due to non-reported or inadequate randomisation and allocation concealment methods [18], [19]. All but one trial were at high risk of performance bias as participants and personnel were reported to have been unblinded [22]. All studies were at low risk of attrition bias as loss to follow-up was minimal and generally well accounted for.

**Table 4:** Risk of bias assessment of included trials.

	Selection bias (randomsequence generation &allocation concealment)	Performance bias (blinding of participants and personnel)	Detection bias (blindingof outcome assessors)	Attrition bias (incomplete outcome assessment)
Harling 2005 [17]	Low risk (block randomisation,factorial design, use of sealedenvelopes)	High risk (unblinded)	High risk (unblinded)	Low risk (>83% follow-up)
Saugstad 1998 [18]	High risk (Quasi randomisation,allocation concealment notreported)	High risk (unblinded)	High risk (unblinded)	Low risk (<10% from each group lost to follow-up)
Lundstrøm 1995 [19]	Unclear risk (not reported)	Unclear risk (not reported)	Unclear risk (not reported)	Low risk (>80% follow-up, withdrawals reported)
Vento 2009 [20]	Low risk (computer generatedsequence, use of sealedenvelopes)	High risk (unblinded)	High risk (unblinded)	Low risk (80% follow-up, withdrawals reported)
Wang 2008 [21]	Low risk (block randomisation,use of sealed envelopes	High risk (unblinded)	High risk (unblinded)	Low risk (>95% follow-up)
Rabi 2011 [22]	Low risk (computer generatedsequence, use of sealedopaque envelopes)	Low risk (biostatistician, data collector, resuscitation team,and carers blinded)	High risk (investigator not blinded)	Low risk (>80% follow-up)

### Effect Size Estimates

#### Primary outcomes

**Figure 2:**
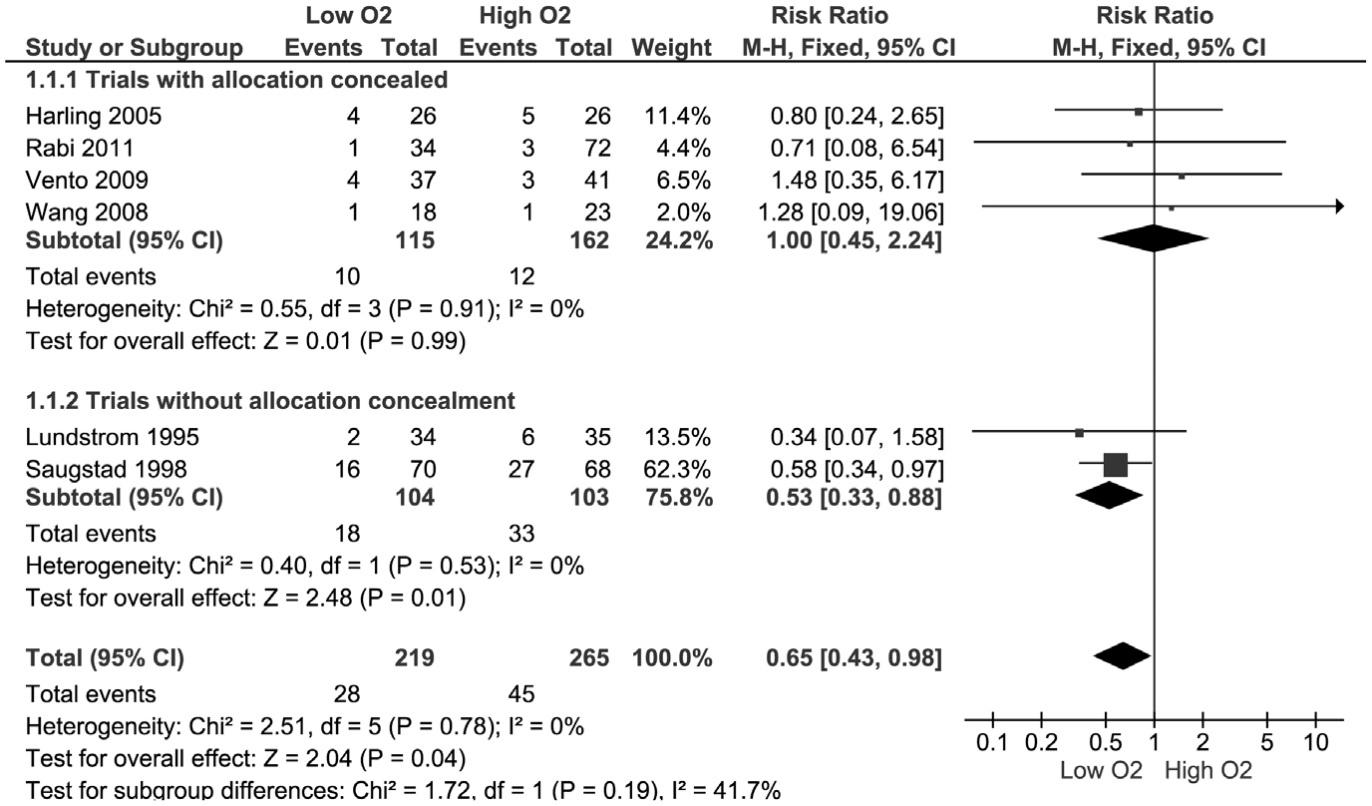
Meta-analysis of effect on mortality prior to hospital discharge.

**Figure 3:**
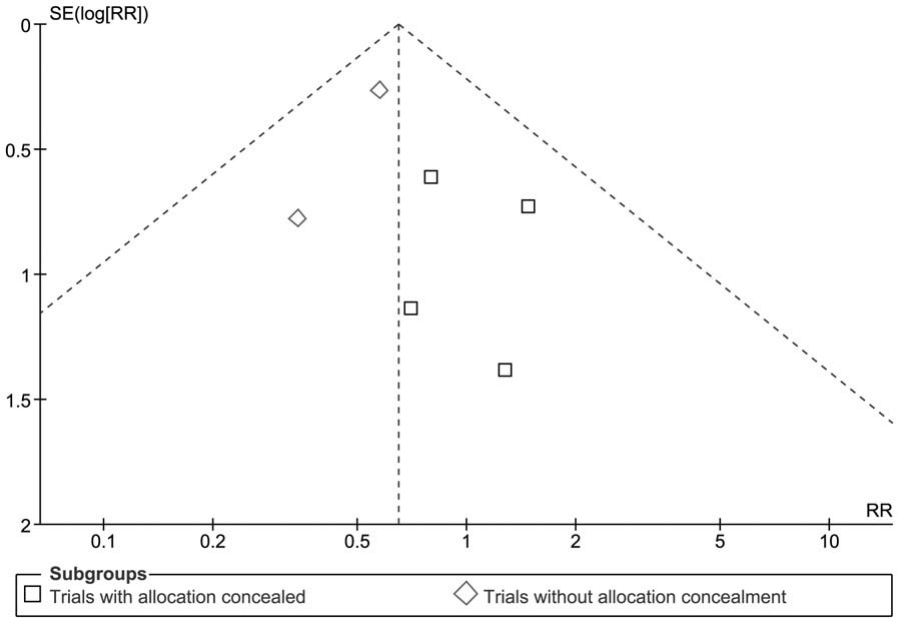
Funnel plot of effect on mortality prior to hospital discharge.

None of the trials individually found a statistically significant effect but meta-analysis of data from all six trials found a borderline statistically significant reduction in mortality in lower oxygen group: pooled RR 0.65 (95% CI 0.43, 0.98), RD: −0.07 (95% CI −0.13, −0.00). There was not any statistical evidence of heterogeneity (I^2^ = 0%) or funnel plot asymmetry (see figures 2 and 3).

A sensitivity analysis restricted to the four randomised controlled trials with adequate allocation concealment did not find a statistically significant difference: pooled RR 1.00 (95% CI 0.45, 2.24), RD: −0.00 (95% CI −0.06, −0.06). None of the trials assessed neuro-developmental outcomes.

#### Secondary outcomes

The included studies did not provide consistent evidence of any statistically significant effects on Apgar score assessment up to 10 minutes after birth (table 5). The effects on the other secondary outcomes are described in tables 6 and 7.

**Table 5:** Apgar scores (time after birth): median and inter-quartile range (if available).

Study	*1 minute*		*5 minutes*		*10 minutes*	
	*high*	*low*	*high*	*low*	*high*	*low*
Harling2005 [17]	6.5(1–10)	5(2–9)	8(3–10)	8(3–10)	NR	NR
Saugstad 1998 [18]	4.4(1.6)*	4.3(1.9)*	7.3(1.9)*	7.3(1.7)*	7.8(1.8)*	8.0(1.4)*
Lundstrøm 1995 [19]	8(3–10)	8(4–10)	10(6–10)	10(8–10)	NR	NR
Vento 2009 [20]	6(2–8)	5(2–7)	8(5–9)	8(5–9)	NR	NR
Wang 2008 [21]	4	5	9	8	7	8
Rabi 2011 [22]	7	6	8	7	NR	NR

NR = not reported, * mean (standard deviation).

**Table 6:** Neonatal morbidity outcomes.

Outcome	N = trials(participants)	Typical RR(95% CI)
**Endotracheal intubation**	3 (225)	0.97 (0.72, 1.29).
**Surfactant replacement**	3 (188 )	1.03 (0.68, 1.58).
**Reached target oxygen saturation by:**		
*3 minutes*	1 (106)	0.42 (0.10, 1.83)
*5 minute*	2 (184)	0.94 (0.80, 1.11)
*8 minutes*	1 (106)	0.91 (0.47, 1.77)
*10 minutes*	3 (231)	0.96 (0.84, 1.11)
**CLD or BPD**	3 (223 )	0.86 (0.62, 1.18)
**ROP**	3 (199)	0.68 (0.24, 1.96)
**NEC**	3 (199)	1.74 (0.42, 7.20)
**Severe IVH**	4 (240)	1.50 (0.71, 3.15)

**Table 7:** Duration of care and admission.

Duration of:	N = Trials(participants)	WMD (95% CI) days
**Mechanical ventilation**	2 (147)	−1.4 (−6.6, 3.9)
**Supplemental oxygen**	1 (78)	−16 (not reported)
**Hospital stay**	2 (180)	−5.0 (−6.9, −3.2)

### Analysis of Subgroups or Subsets

1. Very preterm infants: All of the trials except one recruited predominantly very preterm infants [18]. Exclusion of this trial from the meta-analysis resulted in a change in the pooled risk estimate of the primary outcome [RR: 0.77 (95% CI 0.38, 1.54), RD: −0.11 (95% CI −0.26, 0.04)].
2. Trials undertaken in low or middle-income countries: Only one (quasi-randomised) trial was undertaken in low or middle-income settings (predominantly India, Philippines, and Egypt) [18]. This trial found a statistically significant reduction in the primary outcome in the low oxygen group: RR: 0.58 (95% CI 0.34, 0.97), RD: −0.17 (95% CI −0.32, −0.02).

## Discussion

These data do not provide strong evidence that using lower versus higher oxygen concentrations for delivery room transition support for preterm infants confers important benefits or harms. Although the meta-analysis of all trial data suggested a substantial and statistically significant reduction in mortality, this effect disappeared when only the four trials that concealed allocation were included. This finding emphasises the potential contribution of methodological design issues, particularly lack of allocation concealment, to systematic bias in trials and meta-analyses. Empirical evidence exists that quasi-randomised trials and randomised trials with inadequate concealment of allocation tend to over-estimate effects compared with randomised trials with adequately concealed allocation [25]. Systematic reviews should explore this potential source of bias even in the absence of statistical heterogeneity. It is worth noting that most of the trials included in systematic reviews of different levels of oxygen use for resuscitation of term infants are quasi-randomised [5], [6]. Given the impact that these reviews have had on policy and practice internationally, further exploratory analyses of the potential impact of these trials on over-estimating the effect size is merited.

The available trial data provide limited evidence of the effects on other outcomes. Most importantly, there are not any published data on longer term neuro-developmental outcomes. Assessing the effects on disability and impairment, including cognition, is essential if these trials are to be used to inform policy and practice since delivery room interventions for preterm infants have the potential to have competing effects, that is, they may reduce mortality but with a consequent increase in the risk of disability.

The included trials reported the secondary outcomes, mostly severe neonatal morbidity, inconsistently. Meta-analyses did not detect any statistically significant effects on the incidence of CLD/BPD, ROP, NEC or severe IVH. However, these meta-analyses generally only included data from between two and four trials and the wide 95% CI around the pooled RR estimates do not exclude modest but plausible effect sizes. Some limited data suggested that infants in the lower oxygen group have a shorter hospital stay but this finding should be interpreted with caution as only two trials reported this outcome and the sample size was small.

None of the trials found an effect of lower versus higher oxygen concentration on the Apgar score up to 10 minutes after birth. However, the importance of this outcome measure is uncertain. Firstly, clinicians interpret the clinical signs that contribute to this score subjectively, variably, and inaccurately, especially when applied to preterm infants [26], [27]. Secondly, these assessments are made under stressful conditions and with assessors generally having knowledge of the intervention. It is possible that assessors may have been biased in determining Apgar scores based on prior views on the need for higher concentrations of oxygen for transition support. Thirdly, although the Apgar score is reported as an independent outcome measure, clinicians in these trials generally adjusted the oxygen concentration in response to components of the Apgar score, particularly colour and heart rate. It is likely that the mean Apgar scores at 5 and 10 minutes after birth will have been affected not only by the initial oxygen concentration used but also by subsequent modifications of concentration titrated to clinical response.

### Targeted Oxygen Saturation

In the three most recent of the included trials, the investigators titrated the delivered oxygen concentration to the infant’s target oxygen saturation measured using pulse oximetry [20]–[22]. These trials did not find evidence that the proportion of infants reaching the lower bound of the target saturation at 5 or 10 minutes after birth (saturation generally 80% and 90% respectively) differed between the groups. Most infants allocated to the low oxygen concentration groups in the trials received higher concentrations of oxygen (generally 30% to 60%) in response to clinical and pulse oximetry measures. Similarly, most infants in the higher oxygen concentration groups which pre-specified titrating oxygen delivery to pulse oximetry received lower concentrations by 5 or 10 minutes after birth. These trials did not find significant differences between the groups in the proportion of infants who were hyperoxic (saturation generally >95%) at 5 to 10 minutes after birth. In two earlier trials, pulse oximetry was measured in a convenience subset of participants but the readings were not used in clinical assessment or as indicators to titrate oxygen delivery [18], [19]. In these trials, and in the subgroup of infants in the fixed high oxygen group of another trial [22], the median pulse oximetry readings at 5 or 10 minutes after birth were statistically significantly higher in the high oxygen group.

These finding support the international consensus recommendations to use a blender to titrate oxygen delivery to pulse oximetry in order to avoid hypoxia and hyperoxia during delivery room transition support for very preterm infants [1], [2]. While this has been shown to be feasible, adoption into standard clinical practice has been variable [28]. Currently, fewer than half of neonatal care centres in the UK or Australasia use this approach [29], [30].

The applicability of these recommendations in some low- or middle-income countries is limited by resource availability, specifically lack of oxygen blending equipment [31]. Most of the trials included in this review were conducted in high income countries. One international trial was conducted in several low- and middle-income countries, but the total number of participants from these settings was small and the trial was quasi-randomised and subject to allocation bias [18]. Furthermore, since infants of birth weight <1000 g were not eligible to participate in this trial, the preterm infants were likely to have been more mature that those in the other trials where most infants were very preterm. This difference may have been another contributing factor to the different effect size estimates between the trials. This relative paucity of data from low- and middle-income settings is striking given that almost all neonatal deaths occur in low-income and middle-income countries and that more than one-quarter of deaths are due directly to preterm birth [32].

### Limitations

The main limitation of this review is that few trials were identified and the total number of participants (484) and events is insufficient to detect modest but plausible effect sizes on important outcomes including mortality. Based on the outcomes data in this review (15% mortality rate in controls, pooled risk ratio 0.65), a trial would need 1650 participants to detect this effect with 90% power and type 1 error rate of 5%.

A second limitation is that the *a priori* definition of “low” oxygen concentration includes levels up to 50%. Although most of the included trials compared initial oxygen concentrations of <30% versus >80%, one trial used 50% as the lower concentration versus 100% as the higher. A post hoc exclusion of data from this trial does not affect the effect size estimates. Similarly, exploratory analyses of trials that used a fixed concentration of oxygen compared with trials that allowed titration in response to clinical and pulse oximetry did not change effect size estimates.

### Conclusion

The existing trial data are insufficient to determine how using lower versus higher concentrations of oxygen for delivery room respiratory support affects important outcomes for preterm infants. Although the overall pooled estimate suggests that using lower concentrations of oxygen reduces mortality, this is likely to be an over-estimate of effect size due to allocation bias in quasi-randomised trials. Further large, good-quality randomised controlled trials are needed to resolve this uncertainty.

These may assess different strategies depending on the clinical setting. In high-resource settings, it is likely that clinicians will wish to use pulse oximetry to titrate oxygen administration from either a lower or higher starting point using an oxygen blender. In other settings, particularly in low-and-middle-income countries when pulse oximetry or oxygen blender technology is not readily available, it may be more appropriate to undertake a pragmatic trial of set concentrations (air versus 100% oxygen). Investigators should aim to ensure the participation of very preterm infants as well as infants with evidence of compromise at birth so that subgroup analyses for these populations at high risk of neonatal mortality and morbidity can be planned. The trials should aim to assess important objective outcomes, principally mortality and long-term disability and development.

## Supporting Information

Appendix S1 Search strategy.
(DOCX)

Table S1 PRISMA statement.
(DOC)

Table S2 Excluded studies.
(DOCX)
