# Supplementary material for: Lower versus Higher Oxygen Concentration for Delivery Room Stabilisation of Preterm Neonates: Systematic Review
Source: PLoS One. 2012 Dec 20;7(12):e52033. doi: 10.1371/journal.pone.0052033 (PMC3527365; doi:10.1371/journal.pone.0052033)
Supplement: Appendix S1 — Search strategy. (DOCX) [file pone.0052033.s001.docx]

**Lower versus higher oxygen concentration for delivery room stabilisation of preterm**

**neonates: systematic review**

**Appendix S1**

**Literature search strategies**

**MEDLINE**

OvidSP <http://ovidsp.ovid.com/>

1946 to February Week 1 2012

Searched on 14^th^ February 2012. 2932 records were retrieved. The Cochrane highly sensitive search strategy for identifying randomized trials in MEDLINE (sensitivity-maximizing version) was used to limit retrieval to clinical trials (lines 33-43).[1]

1 exp Infant, Newborn/ (457602)

2 Premature Birth/ (4037)

3 (neonat$ or neo nat$).ti,ab. (165287)

4 (newborn$ or new born$ or newly born$).ti,ab. (113391)

5 (preterm or preterms or pre term or pre terms).ti,ab. (36238)

6 (preemie$ or premie or premies).ti,ab. (95)

7 (prematur$ adj3 (birth$ or born or deliver$)).ti,ab. (9783)

8 (low adj3 (birthweight$ or birth weight$)).ti,ab. (20880)

9 (lbw or vlbw or elbw).ti,ab. (4178)

10 infan$.ti,ab. (284397)

11 (baby or babies).ti,ab. (44352)

12 exp Respiratory Distress Syndrome, Newborn/ (12724)

13 or/1-12 (725808)

14 Oxygen/ (124457)

15 Oxygen Inhalation Therapy/ (10663)

16 oxygen$.ti,ab. (289146)

17 14 or 15 or 16 (348325)

18 exp Resuscitation/ (66246)

19 exp Respiration, Artificial/ (53419)

20 resuscitat$.ti,ab. (36080)

21 ventilat$.ti,ab. (103590)

22 respirat$.ti,ab. (293038)

23 breath$.ti,ab. (73318)

24 (stabilis$ or stabiliz$).ti,ab. (141103)

25 transition$.ti,ab. (157240)

26 Delivery Rooms/ (1001)

27 Birthing Centers/ (457)

28 ((delivery or labo?r or birth$ or maternity) adj3 room$).ti,ab. (1606)

29 ((delivery or labo?r or birth$ or maternity) adj3 ward$).ti,ab. (1687)

30 ((delivery or labo?r or birth$ or maternity) adj3 (unit or units or centre$ or center$)).ti,ab. (3873)

31 or/18-30 (751923)

32 13 and 17 and 31 (9355)

33 randomized controlled trial.pt. (318743)

34 controlled clinical trial.pt. (83442)

35 randomized.ab. (223980)

36 placebo.ab. (128186)

37 drug therapy.fs. (1499563)

38 randomly.ab. (162018)

39 trial.ab. (231031)

40 groups.ab. (1070318)

41 or/33-40 (2779585)

42 exp animals/ not humans/ (3660845)

43 41 not 42 (2359252)

44 32 and 43 (2932)

**Key**

/ = indexing term (MeSH heading)

exp = exploded MeSH heading

$ = truncation

.ti,ab. = terms in either title or abstract fields

adj3 = terms within three words of each other (any order)

? = wildcard - substitute for 1 or no characters, eg: labo?r retrieves labor or labour

.pt.= terms in the publication type field

.fs.= floating subheading

**MEDLINE In-Process & Other Non-Indexed Citations**

OvidSP <http://ovidsp.ovid.com/>

February 13, 2012

Searched on 14^th^ February 2012. 188 records were retrieved.

1 exp Infant, Newborn/ (0)

2 Premature Birth/ (0)

3 (neonat$ or neo nat$).ti,ab. (5145)

4 (newborn$ or new born$ or newly born$).ti,ab. (2935)

5 (preterm or preterms or pre term or pre terms).ti,ab. (1519)

6 (preemie$ or premie or premies).ti,ab. (2)

7 (prematur$ adj3 (birth$ or born or deliver$)).ti,ab. (308)

8 (low adj3 (birthweight$ or birth weight$)).ti,ab. (721)

9 (lbw or vlbw or elbw).ti,ab. (182)

10 infan$.ti,ab. (8790)

11 (baby or babies).ti,ab. (1670)

12 exp Respiratory Distress Syndrome, Newborn/ (0)

13 or/1-12 (15739)

14 Oxygen/ (0)

15 Oxygen Inhalation Therapy/ (0)

16 oxygen$.ti,ab. (22927)

17 14 or 15 or 16 (22927)

18 exp Resuscitation/ (0)

19 exp Respiration, Artificial/ (0)

20 resuscitat$.ti,ab. (1439)

21 ventilat$.ti,ab. (3445)

22 respirat$.ti,ab. (11826)

23 breath$.ti,ab. (3309)

24 (stabilis$ or stabiliz$).ti,ab. (18362)

25 transition$.ti,ab. (39256)

26 Delivery Rooms/ (0)

27 Birthing Centers/ (0)

28 ((delivery or labo?r or birth$ or maternity) adj3 room$).ti,ab. (66)

29 ((delivery or labo?r or birth$ or maternity) adj3 ward$).ti,ab. (76)

30 ((delivery or labo?r or birth$ or maternity) adj3 (unit or units or centre$ or center$)).ti,ab. (175)

31 or/18-30 (73784)

32 13 and 17 and 31 (188)

**Key**

/ = indexing term (MeSH heading)

exp = exploded MeSH heading

$ = truncation

.ti,ab. = terms in either title or abstract fields

adj3 = terms within three words of each other (any order)

? = wildcard - substitute for 1 or no characters, eg: labo?r retrieves labor or labour

**MEDLINE Daily Update**

OvidSP <http://ovidsp.ovid.com/>

February 13, 2012

Searched on 14^th^ February 2012. 8 records were retrieved.

1 exp Infant, Newborn/ (329)

2 Premature Birth/ (20)

3 (neonat$ or neo nat$).ti,ab. (178)

4 (newborn$ or new born$ or newly born$).ti,ab. (95)

5 (preterm or preterms or pre term or pre terms).ti,ab. (63)

6 (preemie$ or premie or premies).ti,ab. (0)

7 (prematur$ adj3 (birth$ or born or deliver$)).ti,ab. (7)

8 (low adj3 (birthweight$ or birth weight$)).ti,ab. (28)

9 (lbw or vlbw or elbw).ti,ab. (9)

10 infan$.ti,ab. (273)

11 (baby or babies).ti,ab. (45)

12 exp Respiratory Distress Syndrome, Newborn/ (2)

13 or/1-12 (630)

14 Oxygen/ (120)

15 Oxygen Inhalation Therapy/ (7)

16 oxygen$.ti,ab. (393)

17 14 or 15 or 16 (443)

18 exp Resuscitation/ (46)

19 exp Respiration, Artificial/ (58)

20 resuscitat$.ti,ab. (29)

21 ventilat$.ti,ab. (102)

22 respirat$.ti,ab. (312)

23 breath$.ti,ab. (75)

24 (stabilis$ or stabiliz$).ti,ab. (211)

25 transition$.ti,ab. (260)

26 Delivery Rooms/ (1)

27 Birthing Centers/ (0)

28 ((delivery or labo?r or birth$ or maternity) adj3 room$).ti,ab. (2)

29 ((delivery or labo?r or birth$ or maternity) adj3 ward$).ti,ab. (3)

30 ((delivery or labo?r or birth$ or maternity) adj3 (unit or units or centre$ or center$)).ti,ab. (5)

31 or/18-30 (948)

32 13 and 17 and 31 (8)

**Key**

/ = indexing term (MeSH heading)

exp = exploded MeSH heading

$ = truncation

.ti,ab. = terms in either title or abstract fields

adj3 = terms within three words of each other (any order)

? = wildcard - substitute for 1 or no characters, eg: labo?r retrieves labor or labour

**Embase**

OvidSP <http://ovidsp.ovid.com/>

1974 to 2012 Week 06

Searched on 14^th^ February 2012. 1786 records were retrieved. A search strategy developed by Lefebvre et al to identify randomised trials in EMBASE was used to limit retrieval to clinical trials (lines 37-51).[2]

1 exp infant/ (481957)

2 newborn/ (449914)

3 prematurity/ (61552)

4 premature labor/ (22000)

5 exp low birth weight/ (31824)

6 (neonat$ or neo nat$).ti,ab. (205535)

7 (newborn$ or new born$ or newly born$).ti,ab. (139965)

8 (preterm or preterms or pre term or pre terms).ti,ab. (46174)

9 (preemie$ or premie or premies).ti,ab. (120)

10 (prematur$ adj3 (birth$ or born or deliver$)).ti,ab. (12626)

11 (low adj3 (birthweight$ or birth weight$)).ti,ab. (24928)

12 (lbw or vlbw or elbw).ti,ab. (5128)

13 infan$.ti,ab. (341638)

14 (baby or babies).ti,ab. (56825)

15 neonatal respiratory distress syndrome/ (6566)

16 or/1-15 (1077995)

17 oxygen/ (128692)

18 oxygen therapy/ (17298)

19 oxygen breathing/ (2448)

20 oxygen$.ti,ab. (369101)

21 or/17-20 (430894)

22 resuscitation/ (52172)

23 exp assisted ventilation/ (88498)

24 resuscitat$.ti,ab. (46143)

25 ventilat$.ti,ab. (136525)

26 respirat$.ti,ab. (372422)

27 breath$.ti,ab. (95379)

28 (stabilis$ or stabiliz$).ti,ab. (178891)

29 transition$.ti,ab. (198178)

30 delivery room/ (1419)

31 maternity ward/ (1952)

32 ((delivery or labo?r or birth$ or maternity) adj3 room$).ti,ab. (2108)

33 ((delivery or labo?r or birth$ or maternity) adj3 ward$).ti,ab. (2159)

34 ((delivery or labo?r or birth$ or maternity) adj3 (unit or units or centre$ or center$)).ti,ab. (4798)

35 or/22-34 (989124)

36 16 and 21 and 35 (12491)

37 random$.ti,ab. (695009)

38 factorial$.ti,ab. (18383)

39 crossover$.ti,ab. (41989)

40 cross-over$.ti,ab. (19179)

41 placebo$.ti,ab. (170554)

42 (doubl$ adj blind$).ti,ab. (127893)

43 (singl$ adj blind$).ti,ab. (11797)

44 assign$.ti,ab. (195276)

45 allocat$.ti,ab. (65858)

46 volunteer$.ti,ab. (155191)

47 Crossover Procedure/ (31837)

48 double blind procedure/ (105467)

49 Randomized Controlled Trial/ (299599)

50 single blind procedure/ (14804)

51 37 or 38 or 39 or 40 or 41 or 42 or 43 or 44 or 45 or 46 or 47 or 48 or 49 or 50 (1163293)

52 36 and 51 (2073)

53 animal/ (1678145)

54 exp animal experiment/ (1597716)

55 Nonhuman/ (3789712)

56 (rat or rats or mouse or mice or hamster or hamsters or animal or animals or dog or dogs or cat or cats or bovine or sheep).ti,ab,sh. (4485327)

57 53 or 54 or 55 or 56 (6394349)

58 exp human/ (13015642)

59 human experiment/ (297622)

60 58 or 59 (13017070)

61 57 not (57 and 60) (5082802)

62 52 not 61 (1786)

**Key:**

/ = indexing term (EMTREE heading)

exp = exploded EMTREE heading

$ = truncation

.ti,ab. = terms in either title or abstract fields

adj3 = terms within three words of each other (any order)

? = wildcard - substitute for 1 or no characters, eg: labo?r retrieves labor or labour

.sh. = terms in the EMTREE heading field

**Maternity and Infant Care**

OvidSP <http://ovidsp.ovid.com/>

1971 to January 2012

Searched on 14^th^ February 2012. 1638 records were retrieved.

1 infant.de. (6662)

2 Infant - newborn.de. (22752)

3 Infant - premature.de. (6910)

4 infant - very premature.de. (688)

5 Infant - low birth weight.de. (2332)

6 Infant - very low birth weight.de. (2076)

7 Premature birth - aetiology.de. (715)

8 Infant - small for gestational age.de. (856)

9 (neonat$ or neo nat$).ti,ab. (26805)

10 (newborn$ or new born$ or newly born$).ti,ab. (12543)

11 (preterm or preterms or pre term or pre terms).ti,ab. (14797)

12 (preemie$ or premie or premies).ti,ab. (32)

13 (prematur$ adj3 (birth$ or born or deliver$)).ti,ab. (2689)

14 (low adj3 (birthweight$ or birth weight$)).ti,ab. (7289)

15 (lbw or vlbw or elbw).ti,ab. (1824)

16 infan$.ti,ab. (40533)

17 (baby or babies).ti,ab. (20575)

18 Respiratory distress syndrome.de. (571)

19 or/1-18 (80175)

20 Oxygen.de. (141)

21 Oxygen inhalation therapy.de. (118)

22 oxygen$.ti,ab. (3273)

23 20 or 21 or 22 (3296)

24 Resuscitation.de. (578)

25 Respiration - artificial.de. (469)

26 resuscitat$.ti,ab. (1412)

27 ventilat$.ti,ab. (2861)

28 respirat$.ti,ab. (5179)

29 breath$.ti,ab. (1229)

30 (stabilis$ or stabiliz$).ti,ab. (444)

31 transition$.ti,ab. (1307)

32 Birth centres.de. (410)

33 ((delivery or labo?r or birth$ or maternity) adj3 room$).ti,ab. (631)

34 ((delivery or labo?r or birth$ or maternity) adj3 ward$).ti,ab. (1083)

35 ((delivery or labo?r or birth$ or maternity) adj3 (unit or units or centre$ or center$)).ti,ab. (3131)

36 or/24-35 (14437)

37 19 and 23 and 36 (1638)

**Key**

.de. = indexing term

$ = truncation

.ti,ab. = terms in either title or abstract fields

adj3 = terms within three words of each other (any order)

? = wildcard - substitute for 1 or no characters, eg: labo?r retrieves labor or labour

**CINAHL**

via EBSCO

Inception to 10^th^ February 2012

Searched on 14^th^ February 2012. 1257 records were retrieved.

| [Search ID#](javascript:__doPostBack('ctl00$ctl00$FindField$FindField$historyControl$ReorderHistoryLink','')) | Search Terms | Results |
| --- | --- | --- |
| S35 | S33 not S34  | (1257) |
| S34 | (MH "Animal Studies")  | (23308) |
| S33 | S15 and S19 and S32  | (1284) |
| S32 | S20 or S21 or S22 or S23 or S24 or S25 or S26 or S27 or S28 or S29 or S30 or S31  | (81943) |
| S31 | TI ( (delivery or labo#r or birth* or maternity) N3 (unit or units or centre* or center*) ) OR AB ( (delivery or labo#r or birth* or maternity) N3 (unit or units or centre* or center*) )  | (1967) |
| S30 | TI ( (delivery or labo#r or birth* or maternity) N3 ward* ) OR AB ( (delivery or labo#r or birth* or maternity) N3 ward* )  | (416) |
| S29 | TI ( (delivery or labo#r or birth* or maternity) N3 room* ) OR AB ( (delivery or labo#r or birth* or maternity) N3 room* )  | (514) |
| S28 | (MH "Delivery Rooms+")  | (989) |
| S27 | TI transition* OR AB transition*  | (13818) |
| S26 | TI ( stabilis or stabiliz* ) OR AB ( stabilis* or stabiliz* )  | (6880) |
| S25 | TI breath* OR AB breath*  | (9667) |
| S24 | TI respirat* OR AB respirat*  | (26307) |
| S23 | TI ventilat* OR AB ventilat*  | (15049) |
| S22 | TI resuscitat* OR AB resuscitat*  | (7526) |
| S21 | (MH "Respiration, Artificial+")  | (11624) |
| S20 | (MH "Resuscitation+")  | (17562) |
| S19 | (S16 or S17 or S18)  | (18708) |
| S18 | TI oxygen* OR AB oxygen*  | (16141) |
| S17 | (MH "Oxygen Therapy")  | (2730) |
| S16 | (MH "Oxygen")  | (2954) |
| S15 | S1 or S2 or S3 or S4 or S5 or S6 or S7 or S8 or S9 or S10 or S11 or S12 or S13 or S14  | (87927) |
| S14 | (MH "Respiratory Distress Syndrome+")  | (1309) |
| S13 | TI ( baby or babies ) OR AB ( baby or babies )  | (12433) |
| S12 | TI infan* OR AB infan*  | (35333) |
| S11 | TI ( lbw or vlbw or elbw ) OR AB ( lbw or vlbw or elbw )  | (1076) |
| S10 | TI ( low N3 (birthweight* or birth-weight*) ) OR AB ( low N3 (birthweight* or birth-weight*) )  | (4336) |
| S9 | TI ( prematur* N3 (birth* or born or deliver*) ) OR AB ( prematur* N3 (birth* or born or deliver*) )  | (1426) |
| S8 | TI ( preemie* or premie or premies ) OR AB ( preemie* or premie or premies )  | (148) |
| S7 | TI ( preterm or preterms or pre-term or pre-terms ) OR AB ( preterm or preterms or pre-term or pre-terms )  | (8559) |
| S6 | TI ( newborn* or new-born* or "newly born*" ) OR AB ( newborn* or new-born* or "newly born*" )  | (9379) |
| S5 | TI ( neonat* or neo-nat* ) OR AB ( neonat* or neo-nat* )  | (18781) |
| S4 | (MH "Childbirth, Premature")  | (2614) |
| S3 | (MH "Infant, Premature")  | (8765) |
| S2 | (MH "Infant, Low Birth Weight+")  | (5361) |
| S1 | (MH "Infant, Newborn+")  | (55142) |

**Key**

MH = indexing term (CINAHL heading)

+ =exploded CINAHL heading

* = truncation

TI = words in the title

AB = words in the abstract

“ “ = phrase search

N3 = terms within three words of each other (any order)

# = wildcard - substitute for 1 or no characters, eg: labo?r retrieves labor or labour

**The Cochrane Library**

Wiley <http://onlinelibrary.wiley.com/>

**Cochrane Database of Systematic Reviews (CDSR)**, Issue 1 of 12, January 2012

**Database of Abstracts of Reviews of Effects (DARE)**, Issue 1 of 4, January 2012

**Health Technology Assessment Database (HTA)**, Issue 1 of 4, January 2012

**Cochrane Central Register of Controlled Trials (CENTRAL)**, Issue 1 of 12, January 2012

The above 4 databases were searched on 14^th^ February 2012, via the Cochrane Library.

1201 records were retrieved in total – 98 from CDSR, 3 from DARE, 1 from HTA, 1099 from CENTRAL.

| ID | | Search | Hits |
| --- | --- | --- | --- |
| #1 | | [MeSH descriptor Infant, Newborn explode all trees](http://onlinelibrary.wiley.com/o/cochrane/searchHistory?mode=runquery&qnum=1) | 11425 |
| #2 | | [MeSH descriptor Premature Birth, this term only](http://onlinelibrary.wiley.com/o/cochrane/searchHistory?mode=runquery&qnum=2) | 241 |
| #3 | | [(neonat* or neo NEXT nat*):ti,ab](http://onlinelibrary.wiley.com/o/cochrane/searchHistory?mode=runquery&qnum=3) | 7054 |
| #4 | | [(newborn* or new NEXT born* or newly NEXT born*):ti,ab](http://onlinelibrary.wiley.com/o/cochrane/searchHistory?mode=runquery&qnum=4) | 3469 |
| #5 | | [(preterm or preterms or pre NEXT term or pre NEXT terms):ti,ab](http://onlinelibrary.wiley.com/o/cochrane/searchHistory?mode=runquery&qnum=5) | 4515 |
| #6 | | [(preemie* or premie or premies):ti,ab](http://onlinelibrary.wiley.com/o/cochrane/searchHistory?mode=runquery&qnum=6) | 13 |
| #7 | | [(prematur* NEAR/3 (birth* or born or deliver*)):ti,ab](http://onlinelibrary.wiley.com/o/cochrane/searchHistory?mode=runquery&qnum=7) | 562 |
| #8 | [(low NEAR/3 (birthweight* or birth NEXT weight*)):ti,ab](http://onlinelibrary.wiley.com/o/cochrane/searchHistory?mode=runquery&qnum=8) | 2104 |  |
| #9 | [(lbw or vlbw or elbw):ti,ab](http://onlinelibrary.wiley.com/o/cochrane/searchHistory?mode=runquery&qnum=9) | 657 |  |
| #10 | [infan*:ti,ab](http://onlinelibrary.wiley.com/o/cochrane/searchHistory?mode=runquery&qnum=10) | 15698 |  |
| #11 | [(baby or babies):ti,ab](http://onlinelibrary.wiley.com/o/cochrane/searchHistory?mode=runquery&qnum=11) | 2611 |  |
| #12 | [MeSH descriptor Respiratory Distress Syndrome, Newborn explode all trees](http://onlinelibrary.wiley.com/o/cochrane/searchHistory?mode=runquery&qnum=12) | 858 |  |
| #13 | [(#1 OR #2 OR #3 OR #4 OR #5 OR #6 OR #7 OR #8 OR #9 OR #10 OR #11 OR #12)](http://onlinelibrary.wiley.com/o/cochrane/searchHistory?mode=runquery&qnum=13) | 25328 |  |
| #14 | [MeSH descriptor Oxygen, this term only](http://onlinelibrary.wiley.com/o/cochrane/searchHistory?mode=runquery&qnum=14) | 3756 |  |
| #15 | [MeSH descriptor Oxygen Inhalation Therapy, this term only](http://onlinelibrary.wiley.com/o/cochrane/searchHistory?mode=runquery&qnum=15) | 748 |  |
| #16 | [oxygen*:ti,ab](http://onlinelibrary.wiley.com/o/cochrane/searchHistory?mode=runquery&qnum=16) | 16310 |  |
| #17 | [(#14 OR #15 OR #16)](http://onlinelibrary.wiley.com/o/cochrane/searchHistory?mode=runquery&qnum=17) | 17560 |  |
| #18 | [MeSH descriptor Resuscitation explode all trees](http://onlinelibrary.wiley.com/o/cochrane/searchHistory?mode=runquery&qnum=18) | 3119 |  |
| #19 | [MeSH descriptor Respiration, Artificial explode all trees](http://onlinelibrary.wiley.com/o/cochrane/searchHistory?mode=runquery&qnum=19) | 3974 |  |
| #20 | [resuscitat*:ti,ab](http://onlinelibrary.wiley.com/o/cochrane/searchHistory?mode=runquery&qnum=20) | 1566 |  |
| #21 | [ventilat*:ti,ab](http://onlinelibrary.wiley.com/o/cochrane/searchHistory?mode=runquery&qnum=21) | 10639 |  |
| #22 | [respirat*:ti,ab](http://onlinelibrary.wiley.com/o/cochrane/searchHistory?mode=runquery&qnum=22) | 20364 |  |
| #23 | [breath*:ti,ab](http://onlinelibrary.wiley.com/o/cochrane/searchHistory?mode=runquery&qnum=23) | 8113 |  |
| #24 | [(stabilis* or stabiliz*):ti,ab](http://onlinelibrary.wiley.com/o/cochrane/searchHistory?mode=runquery&qnum=24) | 4425 |  |
| #25 | [transition*:ti,ab](http://onlinelibrary.wiley.com/o/cochrane/searchHistory?mode=runquery&qnum=25) | 1742 |  |
| #26 | [MeSH descriptor Delivery Rooms, this term only](http://onlinelibrary.wiley.com/o/cochrane/searchHistory?mode=runquery&qnum=26) | 44 |  |
| #27 | [MeSH descriptor Birthing Centers, this term only](http://onlinelibrary.wiley.com/o/cochrane/searchHistory?mode=runquery&qnum=27) | 18 |  |
| #28 | [((delivery or labo*r or birth* or maternity) NEAR/3 room*):ti,ab](http://onlinelibrary.wiley.com/o/cochrane/searchHistory?mode=runquery&qnum=28) | 137 |  |
| #29 | [((delivery or labo*r or birth* or maternity) NEAR/3 ward*):ti,ab](http://onlinelibrary.wiley.com/o/cochrane/searchHistory?mode=runquery&qnum=29) | 150 |  |
| #30 | [((delivery or labo*r or birth* or maternity) NEAR/3 (unit or units or centre* or center*)):ti,ab](http://onlinelibrary.wiley.com/o/cochrane/searchHistory?mode=runquery&qnum=30) | 248 |  |
| #31 | [(#18 OR #19 OR #20 OR #21 OR #22 OR #23 OR #24 OR #25 OR #26 OR #27 OR #28 OR #29 OR #30)](http://onlinelibrary.wiley.com/o/cochrane/searchHistory?mode=runquery&qnum=31) | 40438 |  |
| #32 | [(#13 AND #17 AND #31)](http://onlinelibrary.wiley.com/o/cochrane/searchHistory?mode=runquery&qnum=32) | 1208 |  |
| #33 | [(#13 AND #17 AND #31)](http://onlinelibrary.wiley.com/o/cochrane/searchHistory?mode=runquery&qnum=33) | 1201 |  |

NB: #33 restricted to: CDSR, DARE, HTA, CENTRAL databases.

**Key**

MeSH descriptor = indexing term (MeSH heading)

* = truncation

:ti,ab = terms in either title or abstract fields

NEAR/3 = terms within three words of each other (any order)

NEXT = terms are next to each other

***Ongoing trials***

**ClinicalTrials.gov**

<http://www.clinicaltrials.gov/>

Searched on 20^th^ February 2012. 313 studies found in total. 296 using strategy 1 and 75 studies found using strategy 2.

1. Oxygen AND (infant OR infants OR newborn OR newborns OR premature OR prematurity OR neonate OR neonates OR neonatal OR preterm OR preterms OR preemie OR preemies OR premie OR premies OR birthweight OR baby OR babies)

2. oxygen | "Respiratory Distress Syndrome, Newborn"

**metaRegister of Controlled Trials (mRCT)**

<http://www.controlled-trials.com/mrct/searchform>

Searched on 20^th^ February 2012. 543 studies found in total.

(Oxygen AND (infant OR infants OR newborn OR newborns OR premature OR prematurity OR neonate OR neonates or neonatal OR preterm OR preterms OR preemie OR preemies OR premie OR premies OR birthweight OR baby OR babies))

**WHO International Clinical Trials Registry Platform**

<http://apps.who.int/trialsearch/AdvSearch.aspx>

Searched on 20^th^ February 2012. 87 studies found in total. 47 using strategy 1 and 40 using strategy 2.

1. oxygen* in title, clinical trials in children – 47

2. oxygen* in intervention field, clinical trials in children – 40

***Guidelines***

The following websites were searched on 9^th^ March 2012 and records retrieved were browsed for relevance. A total of 24 guidelines in total were found.

**National Guideline Clearinghouse**

<http://www.guideline.gov/>

**National Institute for Health and Clinical Excellence (NICE)**

<http://www.nice.org.uk/>

**Scottish Intercollegiate Guidelines Network (SIGN)**

<http://www.sign.ac.uk/>

**Turning research into practice database (TRIP)**

<http://www.tripdatabase.com/>

**Resuscitation Council (UK)**

<http://www.resus.org.uk/>

**European Resuscitation Council**

<https://www.erc.edu/index.php/mainpage/en/>

**Royal College of Paediatrics and Child Health**

<http://www.rcpch.ac.uk/>

**Royal College of Obstetricians and Gynaecologists**

<http://www.rcog.org.uk/>

**Royal College of Midwives**

<http://www.rcm.org.uk/>

**British Association of Perinatal Medicine**

<http://www.bapm.org/>

**References**

1. Lefebvre C, Manheimer E, Glanville J (2011) Chapter 6: Searching for studies. In: Higgins JPT, Green S, editors. Cochrane Handbook for Systematic Reviews of Interventions Version 5.1.0 (updated March 2011). The Cochrane Collaboration. Available from [www.cochrane-handbook.org](http://www.cochrane-handbook.org).

2. Lefebvre C, Eisinga A, McDonald S, Paul N (2008) Enhancing access to reports of randomized trials published world-wide - the contribution of EMBASE records to the Cochrane Central Register of Controlled Trials (CENTRAL) in The Cochrane Library. Emerg Themes Epidemiol 5:13.
