# Supplementary material for: Lower versus Higher Oxygen Concentration for Delivery Room Stabilisation of Preterm Neonates: Systematic Review
Source: PLoS One. 2012 Dec 20;7(12):e52033. doi: 10.1371/journal.pone.0052033 (PMC3527365; doi:10.1371/journal.pone.0052033)
Supplement: Table S2 — Excluded studies. (DOCX) [file pone.0052033.s003.docx]

| Study | | Title | Reason for exclusion |
| --- | --- | --- | --- |
| Badiee 2011 [[1](#_ENREF_1)] | | Resuscitation of preterm newborn with high concentration oxygen versus low concentration oxygen | Not an RCT (information provided by the author) |
| Bajaj 2005 [[2](#_ENREF_2)] | | Room air vs. 100 per cent oxygen for neonatal resuscitation: a controlled clinical trial | Subgroup data for included preterm infants was unavailable |
| Clark 2006 [[3](#_ENREF_3)] | | Understanding cardiac troponin T in the newborn period | Letter to the editor |
| Davis 2004 [[4](#_ENREF_4)] | | Resuscitation of newborn infants with 100% oxygen or air: a systematic review and meta-analysis | Systematic review – ordered for background reading |
| Dawson 2012 [[5](#_ENREF_5)] | | Managing oxygen therapy during delivery room stabilization of preterm infants | Commentary |
| Dawson 2009 [[6](#_ENREF_6)] | | Oxygen saturation and heart rate during delivery room resuscitation of infants <30 weeks' gestation with air or 100% oxygen | Non-randomised study |
| Escrig 2008 [[7](#_ENREF_7)] | | Achievement of targeted saturation values in extremely low gestational age neonates resuscitated with low or high oxygen concentrations: a prospective, randomized trial | Report of results for a subgroup of infants included in Vento 2009 [[8](#_ENREF_8)] |
| Escrig 2007 [[9](#_ENREF_9)] | | Achievement of target oxygen saturation in extremely low gestational neonates resuscitated with different oxygen concentrations: a prospective randomized clinical trial | Report of results for a subgroup of infants included in Vento 2009 [[8](#_ENREF_8)] |
| Ezaki 2009 [[10](#_ENREF_10)] | | Resuscitation of preterm infants with reduced oxygen results in less oxidative stress than resuscitation with 100% oxygen | Oxygen level for comparison group not specified |
| Finer 2011 [[11](#_ENREF_11)] | SUPPORT trial: Focussing on ROP and BPD. Early CPAP vs. surfactant in extremely preterm infants | Did not compare different levels of O_2_ |  |
| Guay 2011 [[12](#_ENREF_12)] | No evidence for superiority of air or oxygen for neonatal resuscitation: a meta-analysis | Systematic review – ordered for background reading |  |
| Lanka 2010 [[13](#_ENREF_13)] | Initiating preterm resuscitation with less than 100% oxygen | Letter to the editor |  |
| Perlman 2002 [[14](#_ENREF_14)] | Resuscitation - air versus 100% oxygen | Letter to the editor |  |
| Rabi 2008 [[15](#_ENREF_15)] | Room air versus oxygen administration during resuscitation of preterm infants (ROAR study) | Abstract of Rabi 2011 [[16](#_ENREF_16)] |  |
| Rabi 2007 [[17](#_ENREF_17)] | Room air resuscitation of the depressed newborn: a systematic review and meta-analysis | Systematic review – ordered for background reading |  |
| Ramji 2003 [[18](#_ENREF_18)] | Resuscitation of asphyxiated newborns with room air or 100% oxygen at birth: a multicentric clinical trial | Authors were contacted to request information on any preterm infants included but this information was not provided |  |
| Ramji 1993 [[19](#_ENREF_19)] | Resuscitation of asphyxic newborn infants with room air or 100% oxygen | No preterm infants participated (information provided by O.D. Saugstad) |  |
| Saugstad 2008 [[20](#_ENREF_20)] | Resuscitation of newborn infants with 21% or 100% oxygen: an updated systematic review and meta-analysis | Systematic review – ordered for background reading |  |
| Saugstd 2005 [[21](#_ENREF_21)] | Response to resuscitation of the newborn: early prognostic variables | Not an RCT |  |
| Saugstad 2005 [[22](#_ENREF_22)] | Resuscitation of depressed newborn infants with ambient air or pure oxygen: a meta-analysis | Systematic review – ordered for background reading |  |
| Saugstad 2003 [[23](#_ENREF_23)] | Resuscitation of newborn infants with 21% or 100% oxygen: follow-up at 18 to 24 months | Follow-up report of Saugstad 1998 [[24](#_ENREF_24)] – subgroup data for pre-term infants could not be obtained |  |
| Saugstad 2001 [[25](#_ENREF_25)] | Resuscitation of newborn infants with room air or oxygen | Review – ordered for background reading |  |
| Saugstad 2000 [[26](#_ENREF_26)] | Resuscitation of newborn infants with room air or oxygen | Same data as Saugstad 1998 [[24](#_ENREF_24)] |  |
| Saugstad 1998 [[27](#_ENREF_27)] | Resuscitation with room-air or oxygen supplementation | Review – ordered for background reading |  |
| Sola 2006 [[28](#_ENREF_28)] | Oxygen and oxygenation in the delivery room | Letter to the editor |  |
| Tan 2009 [[29](#_ENREF_29)] | Air versus oxygen for resuscitation of infants at birth | Systematic review – ordered for background reading |  |
| Ten 2009 [[30](#_ENREF_30)] | Room air or 100% oxygen for resuscitation of infants with perinatal depression | Review – ordered for background reading |  |
| Vento 2011 [[31](#_ENREF_31)] | Oxygen supplementation in the delivery room: updated information | Ordered for background reading |  |
| Vento 2010 [[32](#_ENREF_32)] | Resuscitation of the term and preterm infant | Ordered for background reading |  |
| Vento 2004 [[33](#_ENREF_33)] | Enhanced oxidative damage in asphyctic newly born infants resuscitated with pure oxygen as compared to room air | No preterm infants recruited |  |
| Vento 2004 [[34](#_ENREF_34)] | Resuscitation of asphyctic neonates with room air or pure oxygen: switch of the gas source during the procedure | No preterm infants recruited |  |
| Vento 2003 [[35](#_ENREF_35)] | Correlation between pCO_2_ and oxidative stress in asphyxiated infants resuscitated with room air or 100 oxygen | No preterm infants recruited |  |
| Vento 1999 [[36](#_ENREF_36)] | Asphyxiated neonates resuscitation with room air (RAR) vs. oxygen | No preterm infants recruited |  |
| Wang 2007 [[37](#_ENREF_37)] | Room air or oxygen for resuscitation of preterm, very low birthweight (VLBW) neonates | Report of results for a subgroup of infants included in Wang 2008 [[38](#_ENREF_38)] |  |
| Zhu 2007 [[39](#_ENREF_39)] | Which is better to resuscitate asphyxiated newborn infants: room air or pure oxygen? | Meta-analysis |  |

1. Badiee Z, Armanian AM (2011) Resuscitation of preterm newborn with high concentration oxygen versus low concentration oxygen. Journal of Isfahan Medical School 29.

2. Bajaj N, Udani RH, Nanavati RN (2005) Room air vs. 100 per cent oxygen for neonatal resuscitation: a controlled clinical trial. JOURNAL OF TROPICAL PEDIATRICS 51: 206-211.

3. Clark SJ (2006) Understanding cardiac troponin T in the newborn period. AMERICAN JOURNAL OF RESPIRATORY & CRITICAL CARE MEDICINE 173: 816-817.

4. Davis PG, Tan A, O'Donnell CPF, Schulze A (2004) Resuscitation of newborn infants with 100% oxygen or air: a systematic review and meta-analysis. LANCET 364: 1329-1333.

5. Dawson JA, Vento M, Finer NN, Rich W, Saugstad OD, et al. (2012) Managing oxygen therapy during delivery room stabilization of preterm infants. JOURNAL OF PEDIATRICS 160: 158-161.

6. Dawson JA, Kamlin COF, Wong C, te Pas AB, O'Donnell CPF, et al. (2009) Oxygen saturation and heart rate during delivery room resuscitation of infants <30 weeks' gestation with air or 100% oxygen. Archives of Disease in Childhood Fetal & Neonatal Edition 94: F87-91.

7. Escrig R, Arruza L, Izquierdo I, Villar G, Saenz P, et al. (2008) Achievement of targeted saturation values in extremely low gestational age neonates resuscitated with low or high oxygen concentrations: a prospective, randomized trial. PEDIATRICS 121: 875-881.

8. Vento M, Moro M, Escrig R, Arruza L, Villar G, et al. (2009) Preterm resuscitation with low oxygen causes less oxidative stress, inflammation, and chronic lung disease. PEDIATRICS 124: e439-449.

9. Escrig R, Arruza L, Izquierdo I, Villar G, Gimeno A, et al. Achievement of target oxygen saturation in extremely low gestational neonates resuscitated with different oxygen concentrations: a prospective randomized clinical trial; 2007 May 5-8; Toronto, Canada.

10. Ezaki S, Suzuki K, Kurishima C, Miura M, Weilin W, et al. (2009) Resuscitation of preterm infants with reduced oxygen results in less oxidative stress than resuscitation with 100% oxygen. Journal of Clinical Biochemistry & Nutrition 44: 111-118.

11. Finer NN (2011) SUPPORT trial: Focussing on ROP and BPD. Early CPAP vs. surfactant in extremely preterm infants. Monatsschrift fur Kinderheilkunde 159(Suppl. 2): 22.

12. Guay J, Lachapelle J (2011) No evidence for superiority of air or oxygen for neonatal resuscitation: a meta-analysis. CANADIAN JOURNAL OF ANAESTHESIA 58: 1075-1082.

13. Lanka K, Cummings JJ (2010) Initiating preterm resuscitation with less than 100% oxygen. JOURNAL OF PERINATOLOGY 30: 366.

14. Perlman JM (2002) Resuscitation - air versus 100% oxygen. PEDIATRICS 109: 347-349.

15. Rabi Y, Nette-Aguirre A, Singhal N. Room air versus oxygen administration during resuscitation of preterm infants (ROAR study); 2008 2-6 May; Honolulu, Hawaii.

16. Rabi Y, Singhal N, Nettel-Aguirre A (2011) Room-air versus oxygen administration for resuscitation of preterm infants: the ROAR study. PEDIATRICS 128: e374-381.

17. Rabi Y, Rabi D, Yee W (2007) Room air resuscitation of the depressed newborn: a systematic review and meta-analysis. RESUSCITATION 72: 353-363.

18. Ramji S, Rasaily R, Mishra PK, Narang A, Jayam S, et al. (2003) Resuscitation of asphyxiated newborns with room air or 100% oxygen at birth: a multicentric clinical trial. INDIAN PEDIATRICS 40: 510-517.

19. Ramji S, Ahuja S, Thirupuram S, Rootwelt T, Rooth G, et al. (1993) Resuscitation of asphyxic newborn infants with room air or 100% oxygen. PEDIATRIC RESEARCH 34: 809-812.

20. Saugstad OD, Ramji S, Soll RF, Vento M (2008) Resuscitation of newborn infants with 21% or 100% oxygen: an updated systematic review and meta-analysis. Neonatology 94: 176-182.

21. Saugstad OD, Ramji S, Rootwelt T, Vento M (2005) Response to resuscitation of the newborn: early prognostic variables. ACTA PAEDIATRICA 94: 890-895.

22. Saugstad OD, Ramji S, Vento M (2005) Resuscitation of depressed newborn infants with ambient air or pure oxygen: a meta-analysis. BIOLOGY OF THE NEONATE 87: 27-34.

23. Saugstad OD, Ramji S, Irani SF, El-Meneza S, Hernandez EA, et al. (2003) Resuscitation of newborn infants with 21% or 100% oxygen: follow-up at 18 to 24 months. PEDIATRICS 112: 296-300.

24. Saugstad OD, Rootwelt T, Aalen O (1998) Resuscitation of asphyxiated newborn infants with room air or oxygen: an international controlled trial: the Resair 2 study. PEDIATRICS 102: e1.

25. Saugstad OD (2001) Resuscitation of newborn infants with room air or oxygen. Seminars in Neonatology 6: 233-239.

26. Saugstad OD, Rootwelt T, Aalen OO (2000) [Resuscitation of newborn infants with room air or oxygen]. TIDSSKRIFT FOR DEN NORSKE LAEGEFORENING 120: 25-28.

27. Saugstad OD (1998) Resuscitation with room-air or oxygen supplementation. CLINICS IN PERINATOLOGY 25: 741-756.

28. Sola A, Deulofeut R (2006) Oxygen and oxygenation in the delivery room. JOURNAL OF PEDIATRICS 148: 564-565.

29. Tan A, Schulze Andreas A, O'Donnell Colm PF, Davis Peter G (2005) Air versus oxygen for resuscitation of infants at birth. Cochrane Database of Systematic Reviews Issue 2. Art. No.: CD002273. DOI: 002210.001002/14651858.CD14002273.pub14651853.

30. Ten VS, Matsiukevich D (2009) Room air or 100% oxygen for resuscitation of infants with perinatal depression. CURRENT OPINION IN PEDIATRICS 21: 188-193.

31. Vento M, Saugstad OD (2011) Oxygen supplementation in the delivery room: updated information. JOURNAL OF PEDIATRICS 158: e5-7.

32. Vento M, Saugstad OD (2010) Resuscitation of the term and preterm infant. Seminars in Fetal and Neonatal Medicine 15: 216-222.

33. Vento M, Juan S, Miguel A, Ana L, Carmen G, et al. (2004) Enhanced oxidative damage in asphyctic newly born infants resuscitated with pure oxygen as compared to room air. PEDIATRIC RESEARCH 55: 455A.

34. Vento M, Juan S, Miguel A, Fernando GS, Jose V, et al. (2004) Resuscitation of asphyctic neonates with room air or pure oxygen: switch of the gas source during the procedure. PEDIATRIC RESEARCH 55: 560A.

35. Vento M, Sastre J, Asensi M, Lloret A, Garcia-Sala F, et al. (2003) Correlation between pCO_2_ and oxidative stress in asphyxiated infants resuscitated with room air or 100 oxygen. PEDIATRIC RESEARCH 53: 404A.

36. Vento M, Garcia-Sala F, Vina J, Asensi M (1999) Asphyxiated neonates resuscitation with room air (RAR) vs. oxygen. PEDIATRIC RESEARCH 45: 890.

37. Wang CL, Leona TA, Rich W, Finer NN. Room air or oxygen for resuscitation of preterm, very low birthweight (VLBW) neonates; 2007 5-8 May; Toronto, Canada.

38. Wang CL, Anderson C, Leone TA, Rich W, Govindaswami B, et al. (2008) Resuscitation of preterm neonates by using room air or 100% oxygen. PEDIATRICS 121: 1083-1089.

39. Zhu J-J, Wu M-Y (2007) [Which is better to resuscitate asphyxiated newborn infants: room air or pure oxygen?]. Zhonghua Er Ke Za Zhi 45: 644-649.
